# Electrocatalytic hydrogen evolution on the noble metal-free MoS_2_/carbon nanotube heterostructure: a theoretical study

**DOI:** 10.1038/s41598-021-83562-w

**Published:** 2021-02-17

**Authors:** Farhad Keivanimehr, Sajjad Habibzadeh, Alireza Baghban, Amin Esmaeili, Ahmad Mohaddespour, Amin Hamed Mashhadzadeh, Mohammad Reza Ganjali, Mohammad Reza Saeb, Vanessa Fierro, Alain Celzard

**Affiliations:** 1 Surface Reaction and Advanced Energy Materials Laboratory, Chemical Engineering Department, Amirkabir University of Technology (Tehran Polytechnic), Tehran, Iran; 2 Department of Chemical Engineering, McGill University, 3610 University Street, Montreal, QC H3A 0C5 Canada; 3 Department of Chemical Engineering, School of Engineering Technology and Industrial Trades, College of the North Atlantic - Qatar, Doha, Qatar; 4 College of Engineering and Technology, American University of Middle East, Egaila, Kuwait; 5 Center of Excellence in Electrochemistry, School of Chemistry, College of Science, University of Tehran, P.O. Box: 14155-6455, Tehran, Iran; 6 CNRS, IJL, Université de Lorraine, 88000 Épinal, France

**Keywords:** Chemistry, Catalysis, Electrocatalysis

## Abstract

Molybdenum disulfide (MoS_2_) is considered as a promising noble-metal-free electrocatalyst for the Hydrogen Evolution Reaction (HER). However, to effectively employ such material in the HER process, the corresponding electrocatalytic activity should be comparable or even higher than that of Pt-based materials. Thus, efforts in structural design of MoS_2_ electrocatalyst should be taken to enhance the respective physico-chemical properties, particularly, the electronic properties. Indeed, no report has yet appeared about the possibility of an HER electrocatalytic association between the MoS_2_ and carbon nanotubes (CNT). Hence, this paper investigates the synergistic electrocatalytic activity of MoS_2_/ CNT heterostructure for HER by Density Functional Theory simulations. The characteristics of the heterostructure, including density of states, binding energies, charge transfer, bandgap structure and minimum-energy path for the HER process were discussed. It was found that regardless of its configuration, CNT is bound to MoS_2_ with an atomic interlayer gap of 3.37 Å and binding energy of 0.467 eV per carbon atom, suggesting a weak interaction between CNT and MoS_2_. In addition, the energy barrier of HER process was calculated lower in MoS_2_/CNT, 0.024 eV, than in the MoS_2_ monolayer, 0.067 eV. Thus, the study elaborately predicts that the proposed heterostructure improves the intrinsic electrocatalytic activity of MoS_2_.

## Introduction

Hydrogen production from the water-splitting process has attracted increasing attention to meet the global energy demand and provide a viable solution to environmental issues^1^. An economical process for hydrogen production is based on a high-performance surface Hydrogen Evolution Reaction (HER) on an appropriate electrocatalyst^2^. Molybdenum and tungsten sulfides have been identified as promising noble-metal-free electrocatalysts, particularly for the HER process^3–6^. The basic concept of HER mechanisms has also been understood through relationships between computational approaches and corresponding experiments^7–15^. Hinnemann et al. proposed an approach based on the Density Functional Theory (DFT) in which they showed that the exposed edges of MoS_2_ sheets are the active sites for the adsorption of hydrogen if the binding free energy of atomic hydrogen to the electrocatalyst is close to zero^9^. Nevertheless, a critical issue for the application of MoS_2_ as an electrocatalyst in electrochemical reactions is attributed to its low electronic conductivity between two neighboring S–Mo–S sheets, bonded by van der Waals (vdW) forces^16^. The resistivity through the basal planes was indeed determined to be 2200 times larger than that parallel to the planes^16^.

There have been basically two proposed ways to improve the MoS_2_ electrocatalyst towards the HER: (1) increasing the density of active sites at the surface of the electrocatalyst; and (2) enhancing the electrical contact at these sites by reducing the number of layers and by placing MoS_2_ on highly conductive substrates, such as carbon-based materials^16^. Consequently, the stacking of MoS_2_ nanosheets with only a few layers perpendicular to a conductive substrate is expected to be an effective electrocatalyst. This can facilitate charge transfer along the edge of the electrode substrate to the active sites with minimal resistance^16^ while suppressing MoS_2_ aggregation at the same time^17,18^. Recent progress in heterostructures based on vdW forces at the atomic level led to new categories of vertical quantum heterostructures with sharp atomic interfaces between materials having different physicochemical properties^19^. Such heterostructures, including two-dimensional (2D) crystalline layers, can provide interfaces with new physical and chemical characteristics that can be potentially employed in certain applications^20,21^. Moreover, these 2D structures, thanks to the presence of strong covalent bonds, can already provide adequate in-plane stability. However, maintaining the stacking of these heterostructures together requires relatively weak vdW interactions^22^.

The design of atomic layers based on vdW heterostructures can be quite challenging when it comes to adjusting their electrocatalytic activity to the HER process, in order to render them more efficient than electrocatalysts based on metals and metal oxides^23,24^. It was found that no electrocatalytic activity was observed for the defect-free basal planes in most 2D layers^24^. However, the intrinsic electrocatalytic properties of the individual layers for a specific reaction can be significantly changed depending on the design of the various heterostructures. Such changes can be due to the electric field created between the different layers of the active electrocatalysts and the respective conductive substrates^19,25,26^. Namely, the vdW stacking of hexagonal boron nitride and graphene makes the corresponding heterostructure a quite active electrocatalyst towards HER despite the inactive sites of each layer^2,23^. In addition, to evaluate the electrochemical catalytic performance of vdW solids, the selection of the layers in the heterostructure and their sequence must be carefully considered. The importance of the stack layer sequence was addressed experimentally when the electrocatalytic activity of the vdW heterostructure of carbon nanotube (CNT) on MoS_2_ showed a higher HER efficiency than the layered structure of MoS_2_ on CNT^27,28^. In addition, a recent study has indicated that, when graphene is placed on top of MoS_2_, a higher electrocatalytic efficiency can be obtained for the HER process in acidic solution compared to the inverse configuration^26^.

Despite the excellent electronic properties of MoS_2_/carbon-derived electrocatalysts for HER, only a few studies have been carried out on the electrochemical reaction pathways of these advanced materials^24,26^. Specifically, a DFT calculation on MoS_2_/graphene complexes indicated that the presence of graphene as an underlayer of MoS_2_ significantly affects the charge density distribution of MoS_2_^24^. In addition, the induced electric field of the MoS_2_/graphene hybrid provides an excess negative charge density to the system, thus improving its HER activity^24^. Furthermore, this sandwich configuration can also make the MoS_2_ basal plane near the thermo-neutral Gibbs free energy change (${\Delta }G_{H}$ ~ 0), facilitating the activation of the MoS_2_ basal plane towards the HER process. Although a few studies have focused on MoS_2_/graphene, no report was found on the use of MoS_2_/CNT as a HER electrocatalyst. In the present study, we used DFT calculations to understand the molecular mechanism of the stacking sequence and subsequent layers of MoS_2_/CNT in the HER process. Namely, the electrocatalytic activity of a new MoS_2_/CNT heterostructure and the effect of MoS_2_ on the structural and electronic characteristics of the CNT substrate for HER were theoretically explored.

## Methodology

The relaxation of the geometry and the calculation of the electronic structure were performed by the DFT approach. The stacked heterostructure of MoS_2_/CNT was investigated as our main system. In addition, an 8 × 8 CNT (a: 14.01 b: 13.91 c: 17.3 Å) containing 224 carbon atoms was applied to match a 4 × 4 MoS_2_ monolayer (a: 12.66 b: 12.66 c: 18.4 Å) comprising 16 molybdenum and 32 sulfur atoms. The lattice mismatch of the MoS_2_ and CNT layers was approximately 5%. In addition, a void space of 15 Å was considered on the Z-axis to ignore possible interactions between the periodic structures. The relaxation process was carried out for both the MoS_2_ and CNT layers, atoms, and cells, and then the binding energy of the heterostructure was computed using the following equation:

1 $$ E_{b} = E_{total} - E_{CNT} - E_{{MoS_{2} }} $$

where $$ E_{total}$$, $$E_{CNT} $$, and $$E_{{MoS_{2} }}$$ refer to the total energy of the MoS_2_/CNT heterostructure, the energy of a single CNT, and the energy of a single-layer of MoS_2_, respectively. It is worth mentioning that the stable heterostructure was such that the total energy of the MoS_2_/CNT heterostructure is lower than the energy of individual CNT and MoS_2_ (see Eq. (1)) Moreover, the DFT method was used based on the Dmol^3^ code with the Generalized Gradient Approximation (GGA) approach in the Materials Studio version 7.0 package^29,30^. Furthermore, the exchange–correlation functional used in this study was based on the work of Perdew, Burke and Ernzerhof (PBE)^31^ with the Gaussian double zeta plus polarization numerical base (DNP) set. To treat the core electrons, DFT semi-core pseudopotentials (DSPPs) were selected. The geometry relaxation and energy computations were chosen with 1 × 10^–5^ Ha, 0.002 Ha/Å, and 0.005 Å for energy, force, and displacement tolerances, respectively. Thanks to Grimme’s semi-empirical dispersion-corrected density functional theory (DFT-D2)^32,33^ for considering weak interactions with high accuracy, we used DFT-D2 instead of the standard PBE functional. Besides, to analyze the characteristics of the electron density difference, the CASTEP code^34^ of plane wave and ultra-soft pseudopotentials^35^ was applied with a plane-wave cutoff energy of 400 eV.

## Results and discussion

### Optimized structures

**Figure 1:**
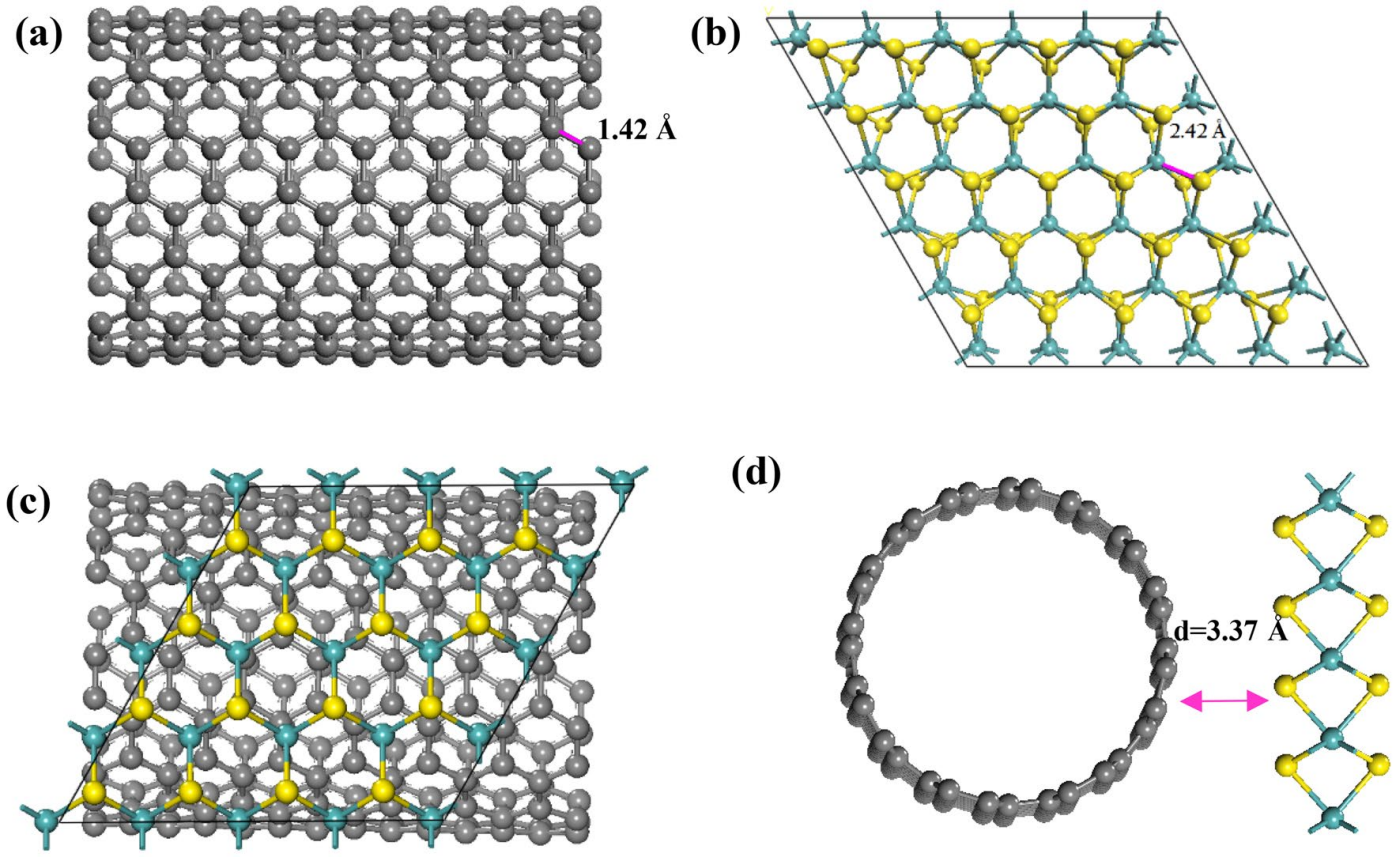
The most stable configurations of: (**a**) CNT; (**b**) MoS_2_; (**c**) top view of MoS_2_/CNT; and (**d**) side view of MoS_2_/CNT. Gray, green and yellow spheres stand for C, Mo and S atoms, respectively.

The optimized configurations of the CNT, MoS_2_, and MoS_2_ deposited on the CNT substrate are shown in Fig. 1. There is a good agreement between the Mo-S bond length of our optimized structure (2.42 Å) and the experimental one (2.41 Å)^36,37^. In addition, a distance of 3.37 Å was optimally achieved between the CNT and the nearest sulfur layer. Since both of the above distances are greater than 1.81 Å (the sum of the covalent radii of carbon and sulfur atoms), vdW forces might be established between the MoS_2_ and the CNT substrate as the main interactions. Furthermore, the average C–C optimized bond length in the CNT nanostructure studied in our work was calculated to be about 1.42 Å, which is in line with the previously reported DFT studies (1.43 Å)^38,39^. Moreover, these results agree with previously published works on graphene/ZnO^40^ and phosphorene/graphene^41,42^ equilibrium distances.

The stability of the MoS_2_/CNT interface was evaluated by computing the binding energy per C atom between MoS_2_ and the CNT substrate. The binding energy obtained was approximately 0.467 eV/atom, similar to the values reported for MoS_2_/graphene^43^ and MoS_2_/N-graphene^44^. Therefore, the value of binding energy can also be considered as a further evidence of the weak vdW interaction forces existing in the MoS_2_/CNT system.

### Density of states (DOS)

**Figure 2:**
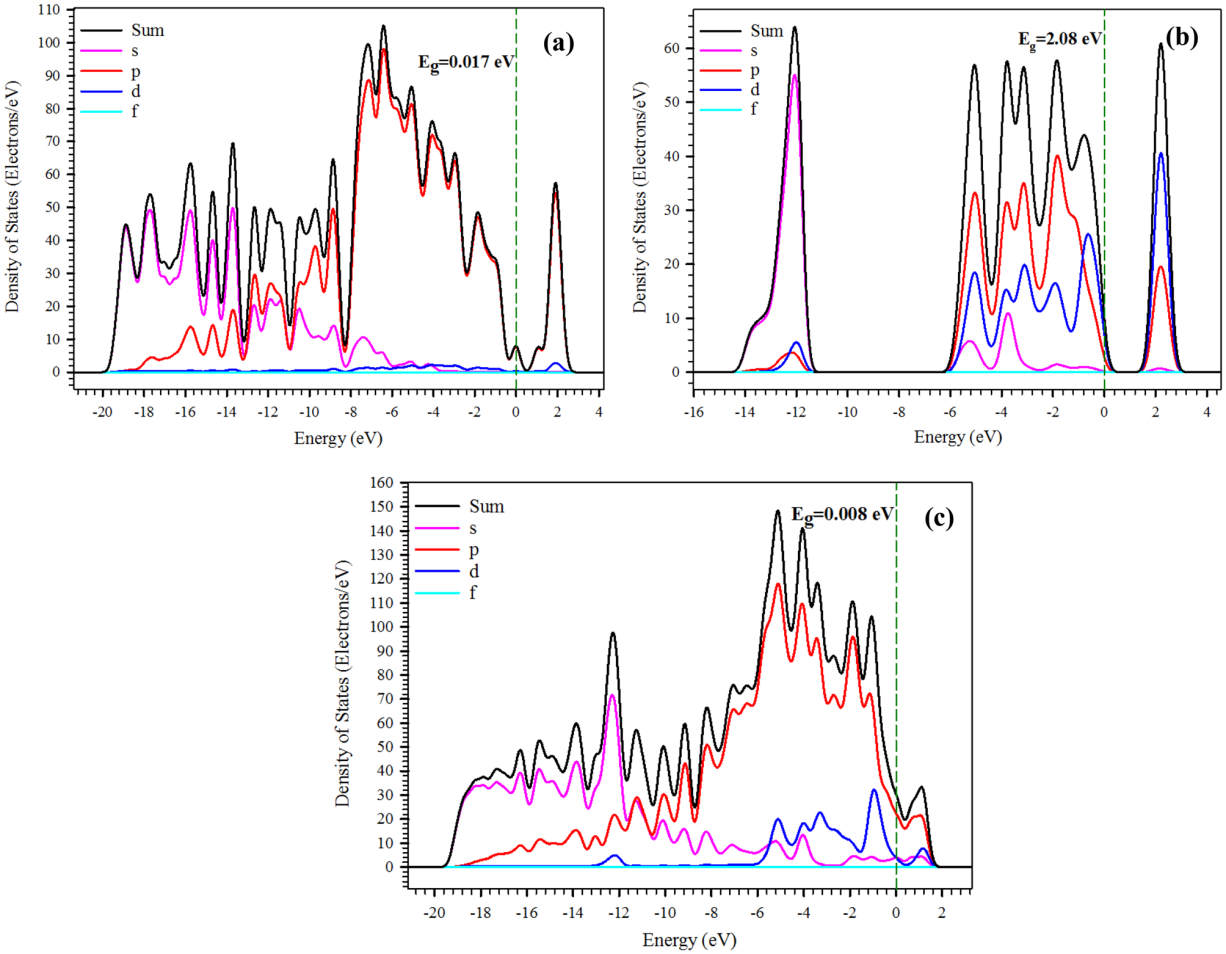
PDOS diagrams for: (**a**) CNT; (**b**) MoS_2_; and (**c**) MoS_2_/CNT. The Fermi energy level is set at zero.

The electronic properties of the system can be determined through the DOS while considering the HOMO–LUMO bandgap energy (*E*_*g*_). This term is the minimum energy required to excite electrons from the valence band to the conduction band. A lower *E*_*g*_ can result in greater system conductivity and hydrogen adsorption capacity. During the formation of the vdW heterostructure, the electronic structure of the CNT on the one hand and of the MoS_2_ monolayer on the other hand were altered near the Fermi energy level. As seen in Fig. 2, the DOS of the MoS_2_/CNT structures was compared to the DOS of the isolated constituent monolayers.

Furthermore, the projected density of states (PDOS) for the C-*p* orbitals in the CNT and the S-*p* and Mo-*d* orbitals in MoS_2_ are shown in Fig. 2a,b. It should be mentioned that CNTs are two dimensional Dirac materials with a linear dispersion near the Fermi energy level^45,46^, as observed in Fig. 2a. In addition, the MoS_2_ monolayer as a semiconductor possesses a bandgap of about 1.8 eV^47^. However, the bandgap obtained for MoS_2_ was calculated to be 2.08 eV in the current simulation (see Fig. 2b). This difference of *E*_*g*_ can be attributed to the unfilled *d*-orbital in the Mo atoms. Thus, semi-local DFT functionals cannot be used to calculate the corresponding *E*_*g*_ correctly, which requires the application of many-body corrections by Green’s function (GW)^48^ or hybrid Heyd, Scuseria, and Ernzerhof (HSE) functionals^49^ to compensate for the bandgap differences of 0.28 eV in the semi-local DFT functionals. However, to reduce computational costs, such bandgap corrections were ignored because the semi-empirical DFT-D2 method can achieve sufficient accuracy for the calculation of structural and electronic properties of the MoS_2_/CNT interface^50^.

**Figure 3:**
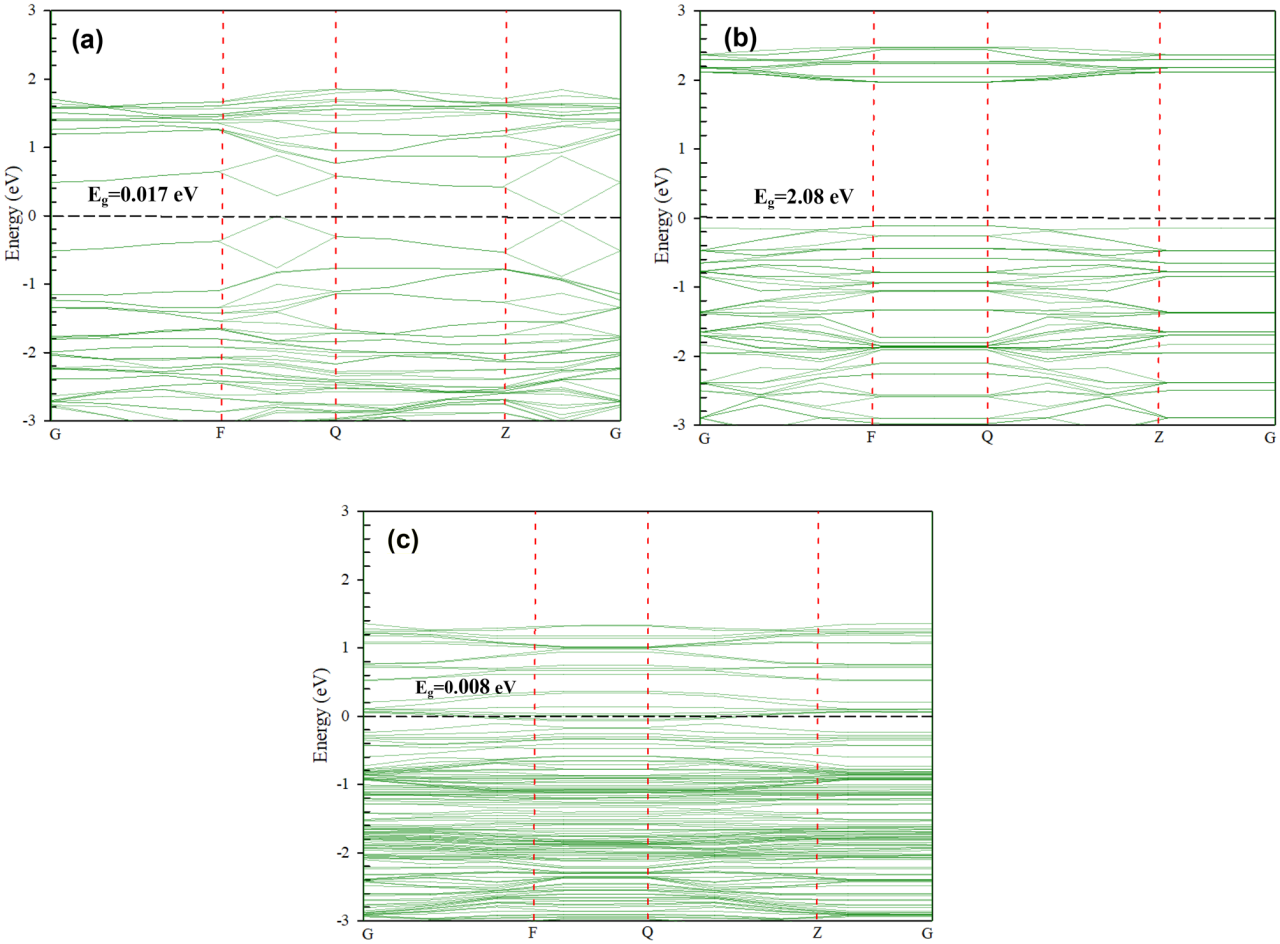
Band structures of: (**a**) CNT; (**b**) MoS_2_; and (**c**) MoS_2_/CNT.

Furthermore, the bottom of the conduction band and the top of the valence band originate mainly from the Mo-*d* orbitals and both the Mo-*d* and S-*p* orbitals, respectively (see Fig. 2b). In addition, it can be seen that the Mo-*d* and S-*p* orbitals were hybridized together at the top of the valence band (see Fig. 2b). It can be concluded from Fig. 2a that the half-filled *p* orbitals perpendicular to the planar structure create the *π* and π* bands in the electronic configuration of CNT. Moreover, at the corner of the Brillouin zone of the CNT, both bonding and antibonding bands touch at a single point near the Fermi energy level. Furthermore, the PDOS of MoS_2_/CNT (see Fig. 2c) is displayed relative to the partial *d*-DOS (blue) of Mo in MoS_2_ and the *p*-DOS (red) of C in the CNT. It can be seen that the Fermi energy level of the MoS_2_/CNT heterostructure is characterized by the Dirac-cone-like characteristic from CNT and a gap-like characteristic from MoS_2_ (see Fig. 3c). Furthermore, the location of PDOSs for CNT is similar to that of MoS_2_/CNT where there is also a remarkable change in the intensity and profile of the PDOSs of MoS_2_.

### Band structure analysis

To evaluate the impact of stacking configurations on the bandgap and electronic properties of the MoS_2_/CNT heterostructure, structural and electronic calculations were performed separately on the freestanding CNT and the MoS_2_ monolayer. As seen in Fig. 3a, the bandgap obtained for an isolated CNT is determined to be 0.017 eV. Besides, a bandgap structure based on a linear Dirac dispersion can be observed for the isolated CNT near the Fermi energy level. The band structure of MoS_2_ is illustrated in Fig. 3b, which shows a direct bandgap of 2.01 eV with the conduction and valence bands positioned at the K point. The resultant bandgap is greater than the reported experimental value (about 1.80 eV)^47^. However, this discrepancy can be corrected by applying the GW approximation technique, which is not the subject of our study.

The band structure of the MoS_2_/CNT heterostructure can be determined by the energy bands of the CNT and MoS_2_. Figure 3c shows the linear dispersion bands of the CNT, which are located in the large energy gap of MoS_2_ while the electronic energy band of the pristine CNT can be found without any major change. However, a significant change near the Fermi energy level can be observed. The results obtained from the band structure can evidently show the efficient interactions between CNT and MoS_2_ that improve the electronic properties of the CNT, the band gap of the heterostructure having dropped to 0.008 eV.

### Determination of charge density and charge transfer

Figure S1 illustrates the variations in the average atomic charge (${\Delta }q$) on the sulfur and molybdenum atoms specified by Hirshfeld’s charge analysis. S1–S4 and M1–M4 represent different positions of sulfur and molybdenum atoms, respectively, in the MoS_2_/CNT heterostructure. The average atomic charge obtained on the MoS_2_ monolayer for S and Mo atoms was calculated to be − 0.114 and 0.229 a.u., respectively. Table S1 summarizes the charges on Mo and S atoms in the MoS_2_/CNT heterostructure. It should be noted that the charge on Mo atoms remains almost unchanged at the value of ∼ 0.229 a.u, whereas it changes negatively and significantly on sulfur atoms.

**Figure 4:**
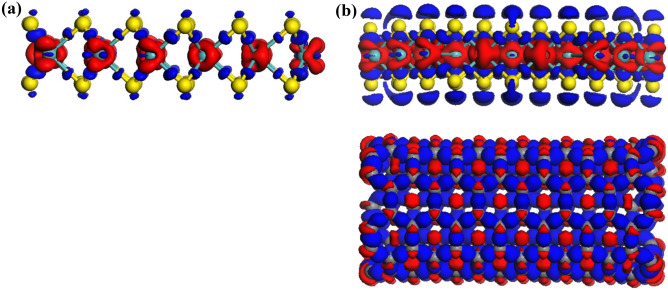
Spatial mapping of charge density differences for: (**a**) a MoS_2_ monolayer; and (**b**) the MoS_2_/CNT heterostructure. Regions of electron accumulation and depletion are denoted by blue and red lobes, respectively. BIOVIA, Dassault Systèmes, Materials Studio, version 7. https://bit.ly/38lRRQR.

Figure 4 illustrates the difference in charge density of the current MoS_2_/CNT system. The charge depletion is observed at the two middle neighboring S and C planes. Moreover, no orbital overlap between the MoS_2_ layer and the CNT can be observed due to the weak vdW interactions between MoS_2_ and the CNT. Besides, the charge transfer, involving the total sum of the Hirshfeld charge populations, was analyzed and computed for the MoS_2_ layer. A negative charge value means that the charge is transferred from the CNT to MoS_2_, while the charge transfer from MoS_2_ to the CNT reflects its positive charge value. Hence, a negative charge value of − 0.051 a.u. obtained for MoS_2_/CNT implies a charge transfer from the CNT to the MoS_2_ monolayer.

### Mechanism of the Hydrogen Evolution Reaction (HER)

The superior electronic structure of MoS_2_/CNT relative to pristine MoS_2_ means that this heterostructure can be used effectively as an electrocatalyst for the HER process. A well-known mechanism of HER, known as Volmer–Heyrovsky or Volmer–Tafel mechanism, can be expressed as follows^51,52^:

2 $$ 2{\text{H}}^{ + } + 2{\text{e}}^{ - } \to 2{\text{H}}^{*} \quad \left( {{\text{Volmer}}} \right) $$

3 $$ {\text{H}}^{*} + {\text{H}}^{ + } \to {\text{H}}_{{2({\text{g}})}} \quad \left( {{\text{Heyrovsky}}} \right) $$

4 $$ {\text{H}}^{*} + {\text{H}}^{*} \to {\text{H}}_{{2({\text{g}})}} \quad \left( {{\text{Tafel}}} \right) $$

According to this mechanism, a hydronium ion first adsorbs on the surface and forms a hydrogen radical. Then, two hydrogen radicals combine and form a hydrogen molecule. Basically, HER activity can be investigated by the adsorption of hydronium ions and the activation energy barrier. The adsorption energy of the hydronium ion adsorbed on MoS_2_ and MoS_2_/CNT monolayer are calculated to be 0.0057 eV and 0.0039 eV, respectively, indicating an easier adsorption of hydronium ion on the MoS_2_/CNT heterostructure than on the MoS_2_ monolayer.

**Figure 5:**
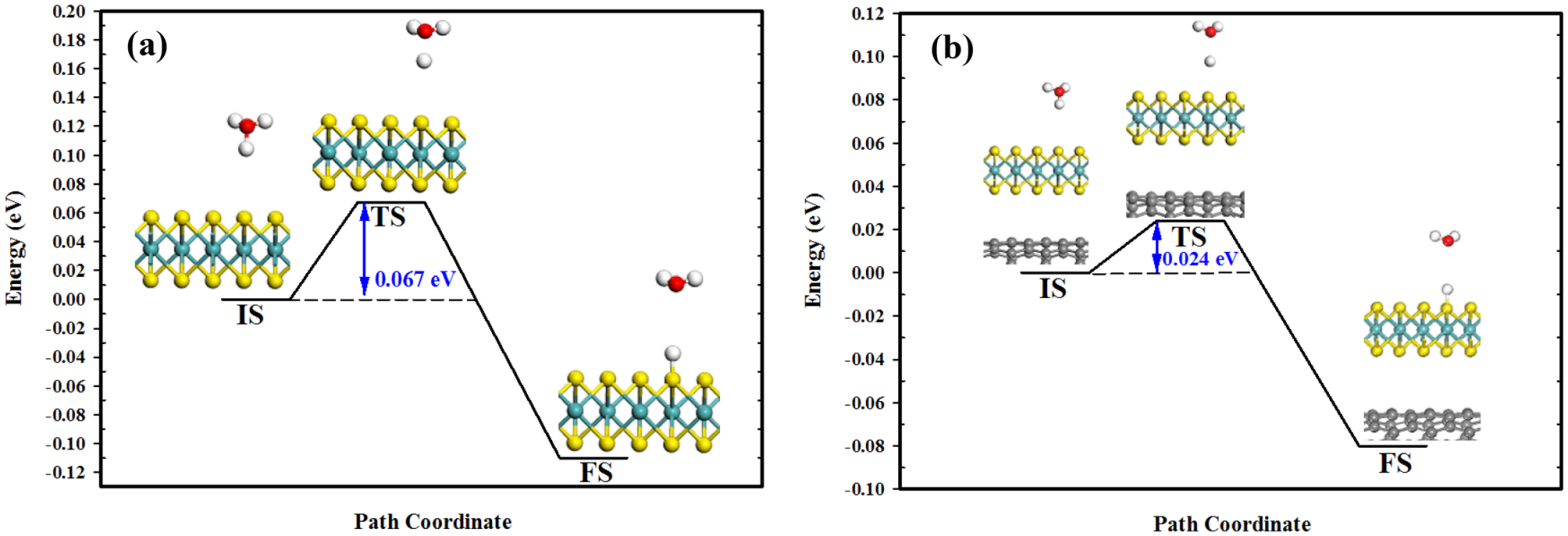
Minimum-energy pathway of the Volmer reaction on: (**a**) MoS_2_; and (**b**) the MoS_2_/CNT heterostructure. IS, TS and FS stand for initial, transition and final states, respectively. BIOVIA, Dassault Systèmes, Materials Studio, version 7. https://bit.ly/38lRRQR.

To characterize the HER activity of the proposed heterostructure, we considered the initial step of the HER process (Volmer reaction) as the rate-determining step (RDS). As illustrated in Fig. 5, the path of minimum energy for the transfer of one of the solvated protons to the MoS_2_ surface in the 4 × 4 supercell consists of three steps: initial, transition and final states (IS, TS and FS, respectively). It can be seen that the adsorption of H atom at each step occurs on the edge of S atom, signifying the electrocatalytic activity of the edge S atoms towards HER. In addition, the energy barrier of MoS_2_/CNT and MoS_2_ monolayer is calculated to be 0.024 eV and 0.067 eV, respectively. This observation might be ascribed to the electron redistribution of the edge S atom after the adsorption of the CNT^53^. From the Hirshfeld charge analysis, the edge S atom of MoS_2_/CNT gains − 0.190 *e* (see Table S1) while that of pristine MoS_2_ acquires − 0.114 *e*. This suggests that due to electrostatic attraction, the edge S atom in MoS_2_/CNT has a more negative charge and thus a greater interaction with the H atom. As a result, it can be seen that the CNT reduces the energy barrier (or the onset potential of HER), thus improving the intrinsic activity of MoS_2_.

**Figure 6:**
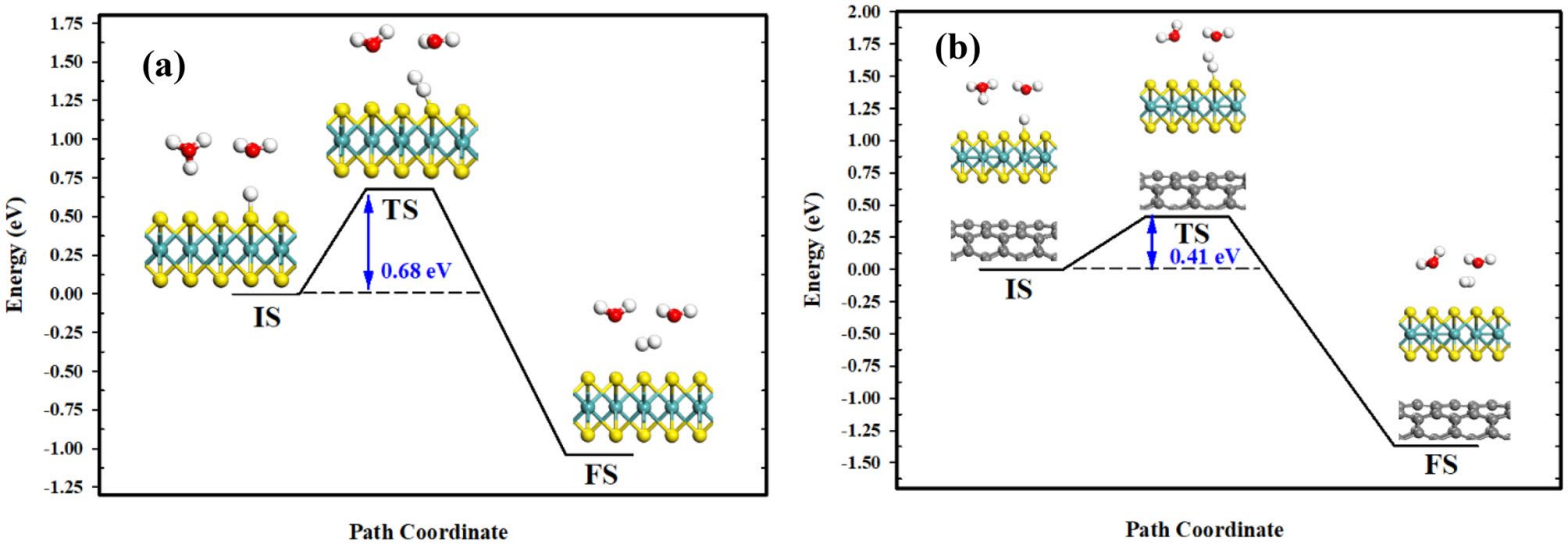
Minimum-energy pathway of the Heyrovsky reaction on: (**a**) MoS_2_; and (**b**) MoS_2_/CNT heterostructure. BIOVIA, Dassault Systèmes, Materials Studio, version 7. https://bit.ly/38lRRQR.

Moreover, the other two primary steps which can be plausible for H_2_ evolution in the second step of the HER process encompass Heyrovsky and Tafel reactions. In the case of Heyrovsky reaction (as the initial step), H_2_ molecule is formed through the reaction of the proton (in the water layer) with an adsorbed hydrogen (see Eq. (3)). Figure 6 displays the estimated minimum-energy paths at two dissimilar structures. It is evident that an adsorbed H atom on a sulfur one reaches an H atom of a hydronium ion in the water layer. This is followed by breaking the adsorbed H from the surface, forming H_2_ molecule within the water layer. In the TS (Fig. 6), the interfacial adsorbed H is separated from the surface and the S–H distance increased from 1.301 Å in the IS to 2.391 Å in the MoS_2_ monolayer. At the same time, the proton from H_3_O^+^ travels toward the separated H atom, forming a molecule with H–H bond lengths of 0.760 Å and 0.756 Å in the MoS_2_ monolayer and MoS_2_/CNT heterostructure, respectively. The evolved H_2_ molecule is detached from the surface in the FS. An activation energy of 0.68 eV was determined for MoS_2_ while such energy barrier drops to 0.41 eV for the MoS_2_/CNT heterostructure (Fig. 6). Thus, the energy barrier is far greater for the Heyrovsky reaction than that for the Volmer reaction, suggesting that the H desorption procedure would be the RDS of the Volmer − Heyrovsky pathway.

**Figure 7:**
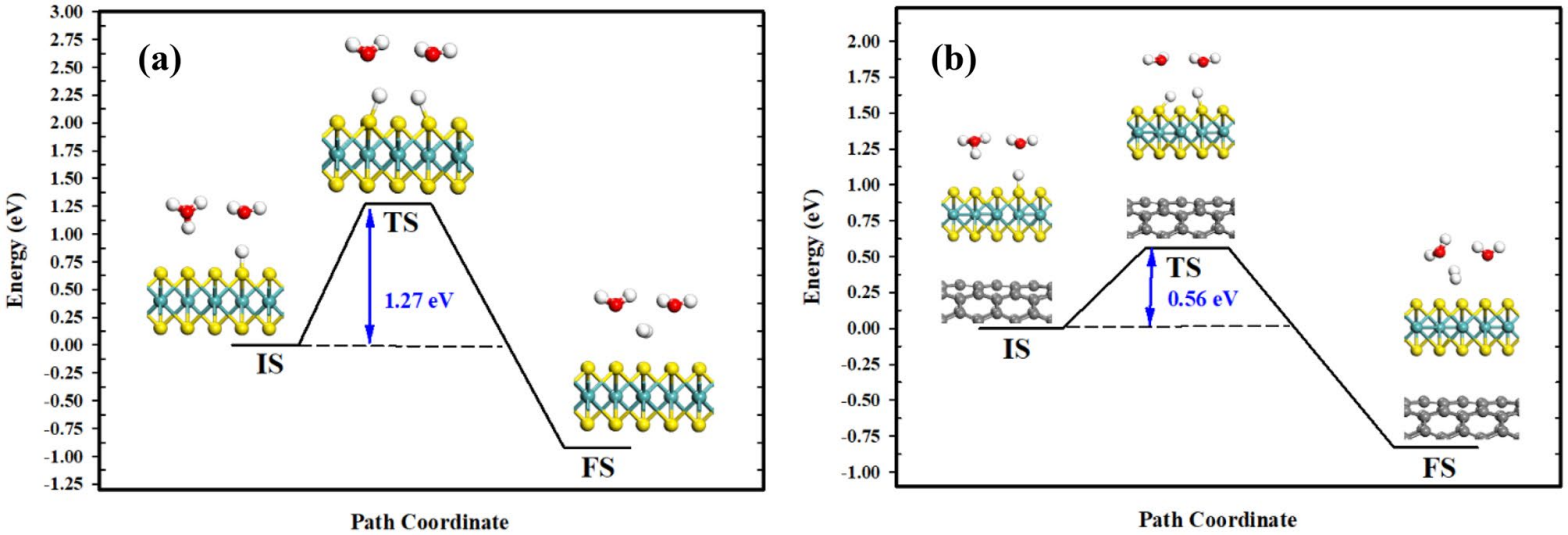
Minimum-energy pathway of the Tafel reaction on: (**a**) MoS_2_; and (**b**) the MoS_2_/CNT heterostructure. BIOVIA, Dassault Systèmes, Materials Studio, version 7. https://bit.ly/38lRRQR.

Furthermore, adjoining H atoms coupling to surface S atoms perform the Tafel reaction on MoS_2_ (see Eq. (4)). Figure 7 depicts the minimum-energy path of direct recombination of two adjoining protons adsorbed on S active sites. Due to the absence of charge transfer over the interface, the entire energies along the path are not amended for possible deviance. In the IS, the distances between the two H atoms are 3.922 Å and 3.817 Å in the MoS_2_ monolayer and MoS_2_/CNT heterostructure, respectively. The two S–H bond lengths in the MoS_2_ monolayer and MoS_2_/CNT heterostructure are 1.309 Å and 1.324 Å, respectively. However, the formed H_2_ molecule undergoes desorption from the surface with a H–H bond length of 0.750 Å for both of the developed structures in the FS. In the MoS_2_ monolayer and MoS_2_/CNT heterostructure, the estimated energy barrier of direct recombination values are 1.27 eV and 0.56 eV, respectively, which are markedly greater than that for the Volmer reaction. Therefore, the RDS in the Volmer–Tafel reaction is the H desorption (Tafel step).

Overall, a comparison of the energy barriers for the Heyrovsky and Tafel reactions on MoS_2_ monolayer and MoS_2_/CNT heterostructure reveals that the Tafel reaction should surmount elevated barriers of 1.27 and 0.56 eV, respectively, while it is possible for the Heyrovsky reaction to proceed more straightforwardly. This indicates more efficiency of the Heyrovsky reaction where the Volmer − Heyrovsky mechanism is the major pathway of HER. In addition, finding from the recent reports shows that HER potentially occurs through the Volmer − Heyrovsky process on the basal plane of 1 T-MoS_2_, 2H-MoS_2_ and Ni-MoS_2_/RGO^52,54,55^.

## Conclusion

The present study theoretically investigates the synergistic electrocatalytic activity of the MoS_2_/CNT heterostructure towards the HER process by applying DFT simulations. The results indicated a weak van der Waals interaction between the CNT and the MoS_2_ monolayer. Moreover, a distance of 3.37 Å was determined between them, and the binding energy per C atom in this system was found to be approximately 0.467 eV. The bandgap structure indicated that the linear Dirac-like dispersion of CNT near the Fermi energy level remains unchanged in the MoS_2_/CNT interface as well. However, it was found that a bandgap around 8 meV was calculated at the Dirac K-point of the CNT in the MoS_2_/CNT interface. Finally, it was confirmed that the presence of CNT can improve the electronic conductivity while reducing the energy barrier in the MoS_2_/CNT heterostructure for the HER process.

## Supplementary Information

Supplementary Information
