# Supplementary material for: Electrocatalytic hydrogen evolution on the noble metal-free MoS2/carbon nanotube heterostructure: a theoretical study
Source: Sci Rep. 2021 Feb 17;11:3958. doi: 10.1038/s41598-021-83562-w (PMC7889931; doi:10.1038/s41598-021-83562-w)
Supplement: Supplementary file 1 — Supplementary Information [file 41598_2021_83562_MOESM1_ESM.docx]

**Supplementary Information**

**Electrocatalytic Hydrogen Evolution on the** **noble metal-free MoS_2_/carbon nanotube heterostructure: A theoretical study**

Farhad Keivanimehr^1^, Sajjad Habibzadeh^1,2*^, Alireza Baghban^1^, Amin Esmaeili^3^, Ahmad Mohaddespour^4^, Amin Hamed Mashhadzadeh^5^, Mohammad Reza Ganjali^5^, Mohammad Reza Saeb^5^, Vanessa Fierro^6^, Alain Celzard^6^

*^1^Surface reaction and advanced energy materials laboratory, Chemical Engineering Department, Amirkabir University of Technology (Tehran Polytechnic), Tehran, Iran*

*^2^ Department of Chemical Engineering, McGill University, 3610 University Street, Montreal, QC H3A 0C5, Canada.*

*^3^Department of Chemical Engineering, School of Engineering Technology and Industrial Trades, College of the North Atlantic - Qatar, Doha, Qatar*

*^4^College of Engineering and Technology, American University of Middle East, Kuwait*

*^5^Center of Excellence in Electrochemistry, School of Chemistry, College of Science, University of Tehran, P.O. Box: 14155-6455, Tehran, Iran*

*^6^Université de Lorraine, CNRS, IJL, 88000 Epinal, France*

**__________________________________**

^*^ Corresponding author. E-mail address: Sajjad.habibzadeh@mail.mcgill.ca (S.Habibzadeh)


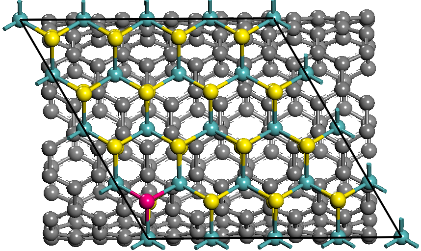

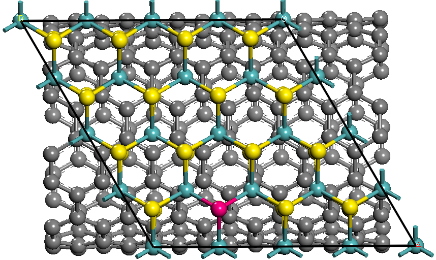

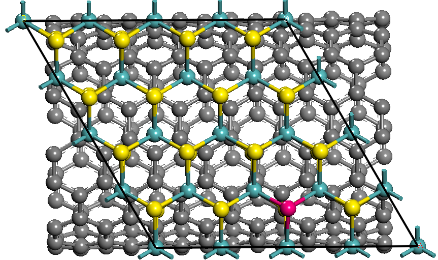

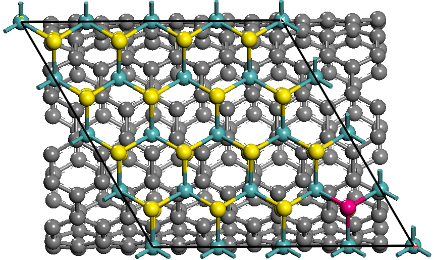

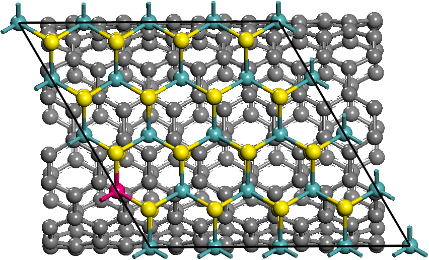

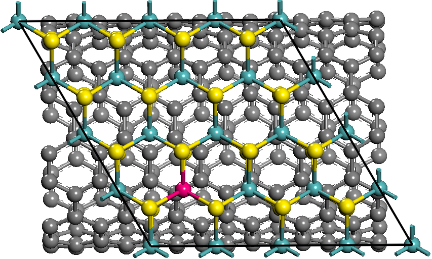

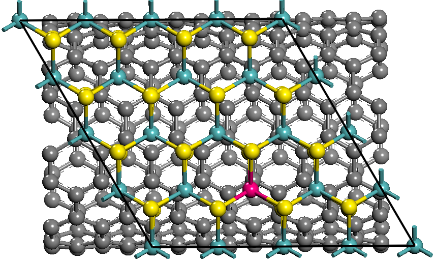

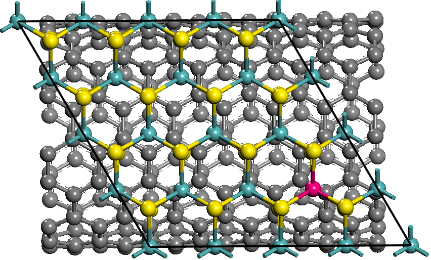

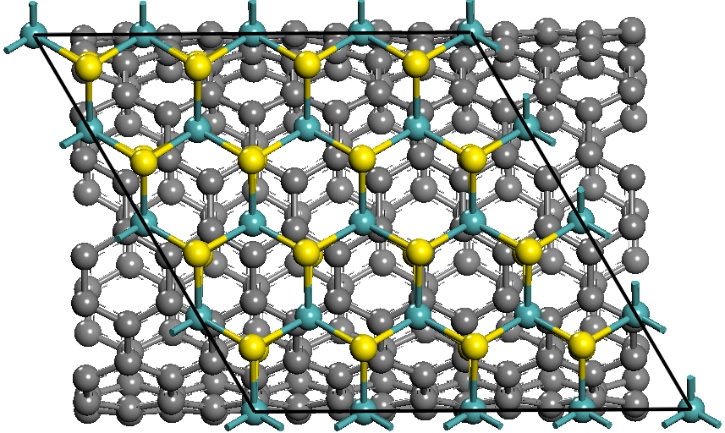


**S1**

**S2**

**S3**

**S4**

**M1**

**M2**

**M3**

**M4**

**Figure S1.** Schematic view of the atomic charge on the MoS_2_/CNT heterostructure for different positions of sulfur (S1-S4) and molybdenum (M1-M4) atoms. BIOVIA, Dassault Systèmes, Materials Studio, version 7. https://bit.ly/38lRRQR

**Table S1**. Atomic charge values on the MoS_2_/CNT heterostructure

| Site location | S1 | S2 | S3 | S4 | M1 | M2 | M3 | M4 |
| --- | --- | --- | --- | --- | --- | --- | --- | --- |
| Charge (a.u.) | -0.190 | -0.142 | -0.140 | -0.191 | 0.221 | 0.229 | 0.229 | 0.220 |
